# Fatherhood experiences: A qualitative approach of cisgender and transgender fathers in assisted reproductive technologies (ART) situation with sperm donation

**DOI:** 10.1016/j.heliyon.2024.e40501

**Published:** 2024-11-16

**Authors:** N. Mendes, L. Woestland, V. Drouineaud, F. Poirier, C. Lagrange, O. Rosenblum, J.-P. Wolf, C. Patrat, J. Brunelle, F. Pommier, D. Cohen, A. Condat

**Affiliations:** a Service de Psychiatrie de l’Enfant et de l’Adolescent, Institut IDEAL, Hôpital Pitié-Salpêtrière, AP-HP, Paris, France; b Service de Biologie de la Reproduction-CECOS, Hôpital Jean Verdier, APHP, Bondy, France; c Service Biologie de la Reproduction–CECOS, Hôpital Cochin, AP-HP, Paris, France; d Équipe d'accueil CLIPSYD EA 4430, connaissance, langage, modélisation (ED 139), Université Paris Nanterre, Nanterre, France; e Institut des Systèmes Intelligents et de Robotiques, Sorbonne Université, Paris, France; f CESP INSERM 1018, ED3C, Université Paris Descartes, Paris, France

**Keywords:** Transgender fathers, Cisgender fathers, Donor sperm insemination, Natural conception, Thematic analysis, Family dynamics, Parenthood

## Abstract

Assisted reproductive technologies have offered new ways and pathways to conceive a child. However, very few is known about insights into the complexities and challenges faced by trans fathers in contemporary parenthood journeys. This qualitative study explores the experiences of transgender and cisgender fathers who conceived children through donor sperm insemination (DSI) and natural conception (NC). The study will examine the recognition and challenges associated with paternal experiences. Seventy-nine interviews (one per child) were conducted with 16 transgender fathers who conceived trough DSI, 15 infertile cisgender fathers who conceived trough DSI, and 17 cisgender fathers who conceived through NC. The study employed thematic analysis and the Five-Minute Speech Sample to assess family dynamics. Results indicate that in the Trans-DSI group, fatherhood was often defined within the social role of masculinity, with some expressing ‘*intranquility*’ (i.e., feelings of unease or insecurity). The Cis-DSI group showed more tranquility but faced challenges regarding biological filiation. The NC group exhibited diverse experiences. Findings suggest that fathers' self-confidence, especially for transgender and non-biologically related fathers, can be challenged. Recognizing these challenges can guide better support for families navigating diverse parenthood journeys.

## 1 Introduction

Despite the widespread recognition of the father's role across different cultures and social structures, fatherhood is an area of limited study, especially in the context of transgender men's experiences of fatherhood. The experiences of cisgender and transgender men as fathers within the context of Assisted Reproductive Technologies (ART) with gamete donation remain a peripheral area of research. Most studies focus on the well-being of children in different parenting configurations [1,2].

In the context of transgender parenthood, there are different possibilities in terms of accessing parental status. Some individuals become parents before beginning the gender transition process, sometimes even before expressing a sense of belonging to a gender different from their assigned sex at birth [[1], [2], [3]].

Another possibility is for transgender men - individuals who were assigned female at birth who identify as male - to carry a pregnancy [4]. In such cases, men suspend hormonal treatment (if they are using testosterone hormone) and can conceive naturally, with artisanal conception, or with assisted reproductive treatment (ART). The impact of testosterone on fertility and the duration of this impact are still uncertain [5]. Therefore, it is crucial to provide information and discuss fertility preservation before starting a transition treatment. There are numerous possibilities for transgender individuals to have a child, including scenarios where a transgender person has a child with another transgender person [6]. However, the main challenges lie in the cost of ART and the varying legal considerations between countries.

In France, a specific couple configuration has been possible since the 1990s: after his transition, a trans man, being in a relationship with a cisgender woman, can benefit from ART with sperm donation. The cisgender women carry the pregnancy. Two studies have examined the outcomes of children in such situations [7,8]. They have not revealed any specific concerns in the psycho-affective development of the children. Most studies of transgender parenthood focus on child family planning or situations outside of ART. In these situations, the methodology used is often phenomenological [[9], [10], [11], [12], [13], [14], [15], [16], [17]]. However, there are no studies that focus specifically on the experiences of transgender fathers, even though these experiences may be important to take into consideration.

Regarding the cisgender context of ART, parents who underwent it to conceive children had significantly more positive perceptions of their children compared to parents who conceived naturally which shows more heterogeneity [18]. One year after childbirth, In Vitro Fertilization/Intracytoplasmic sperm injection (IVF/ICSI) treatment does not appear to be associated with clinical anxiety in fathers [19]. IVF fathers appear to develop a stronger attachment to their child, compared to fathers who conceive naturally [20]. However, some studies have reported negative consequences for father's psychological well-being, such as elevated levels of anxiety or indirect aggression [20,21]. In addition, understanding how families who have used gamete donation experience parenthood is more challenging. In the case of male infertility, using sperm donation is another pathway to building a family. It becomes difficult for them to discuss the donation with the child, as it risks disrupting the father's perceived position as the biological father of the child [22]. However, research suggests that even when the child is informed, there is no negative impact, even at the age of 7–8 years old [23]. In France, a study found that 62 % of the surveyed couples had not informed their children about the gamete donation, and among them, 31 % were considering not discussing it and keeping the origin of the child a secret [24].

The objective of this study is to examine the experiences of transgender and cisgender fathers who have conceived children through donor sperm insemination (DSI) and natural conception (NC). Several authors have distinguished three aspects of parenthood: the exercise of parenthood the practice of parenthood, and the subjective experience of parenthood [25]. The exercise of parenthood refers to the rights and duties of parents towards their children. The practice of parenthood focuses on daily life with the child (i.e., the provision of care and the responses that parents provide). Finally, the experience of parenthood refers to the subjective and emotional dimensions of parenthood. This last dimension is the focus of the current study. The study will 1) examine the recognition and challenges associated with paternal experiences.

## 2 Methods

### 2.1 Design

The study design is a monocentric cross-sectional study over two years. As the recruitment center, the CECOS-Cochin^1^ sperm bank was the first and for a long time the only authorized center in France when the first sperm donation for transgender fathers occurred in 2000. This choice allows us to include fathers who benefited from ART between 2000 and 2016, giving us a fairly large background and children who are old enough. The recruitment covers all regions of France. The study is derived from a larger study originally published by Condat et al. [26]. In this study, we analyzed qualitatively parental expressed emotion towards the child recorded with a specific tool (see below) to explore the recognition and challenges associated with paternal experiences, and 2) provide an analysis of these experiences.

### 2.2 Recruitment of the participants

The protocol was approved by the CERES (*Comité d’Ethique de Recherche en Santé*) of Paris 5 University (Registration number: 2015/31). Our group of interest included only couples with a transman asking for donor sperm insemination (DSI) and referred to as the Trans-DSI group. It comprised 16 transgender fathers and cisgender mothers who conceived through DSI based on the national cohort data available. They had conceived 31 children in total, who represent nearly 60 % (31/52) of all children born in France from a trans father who conceived through DSI. This cohort was formed from a previously published cohort [7] who we contacted to participate in this study. We established two control groups of parents with children that we matched by age and gender with the Trans-DSI group. The first consisted of couples with cis-gender fathers who had previously sought assistance at CECOS-Cochin and were referred to as the Cis-DSI group. Approximately 75 couples undergo progressive pregnancies through DSI at CECOS-Cochin each year, and it is common for these couples to return for second or third donor insemination requests. We recruited them according to the matching opportunities of their previous children. We recruited 15 fathers from cisgender couples who used DSI. The third group, named naturally conceived (NC), included children conceived through sexual intercourse between cisgender parents. These participants were recruited through announcements made during relevant departmental meetings, targeting families of professionals who expressed their consent, as well as through a local public school in the neighborhood. Seventeen fathers were included in this group.

The inclusion criteria encompassed boys and girls under the age of 15 who were born through DSI or conceived naturally in the NC group, and who agreed to participate in a one-day evaluation. The exclusion criteria included (i) poor comprehension of written and/or spoken French, which would impede participants from completing standardized interviews accurately, and (ii) refusal from at least one parent to sign the consent form. All couples included in the study were heterosexual, as DSI was only available for heterosexual couples in France until 2021 [8,27].

### 2.3 Tool

To assess the family dynamics, we utilized the Five-Minute Speech Sample (FMSS), which examines the emotional climate within the family by evaluating attitudes and feelings expressed by a family member toward another family member. This measure, known as "expressed emotions," specifically measures levels of criticism and emotional over-involvement directed towards the child during a recorded session. The instructions given during the session were as follows: "*I would like to hear your thoughts and feelings about (name of family member) in your own words, without any interruption or questions from my side. When I ask you to start, please talk to me for 5 min, describing the kind of person (name of family member) is and how you interact with him/her. Once you begin speaking, I prefer not to ask any questions until the end of the 5 min*"

The fact that the researcher does not intervene during the participant's speech allows us to consider the possibility of rather spontaneous and free associations. No surprise effect is sought during the interview. The interview procedure is explained and participants are allowed to ask any questions they may have beforehand. This ensures that the participant is in an active position and does not give the impression of being put in a difficult position, which could lead to excessive inhibition of discourse. Because the researcher does not intervene during the 5 min, the influence of the researcher's intervention is reduced. Each session with the father was recorded separately for each child by our trained clinician.

An initial analysis of the FMSS interviews using the original tool scoring has already been published for the same cohort [8]. The critical dimension of the FMSS is based on the initial statement, the expressed relationship, any blame or dissatisfaction expressed, while emotional over-involvement includes emotional displays, attitude statements (such as extreme expressions of love), self-sacrifice, overprotection, a lack of objectivity, excessive details about the past, and more than five positive remarks about the child. We found significant differences between Trans-DSI group and Cis-DSI group [19] that motivated to complement these results with an additional qualitative analysis.

### 2.4 Material

We interviewed 48 individual fathers from the three study groups (N = 16 Trans-DSI group; N = 15 Cis-DSI group; N = 17 NC group). Since some fathers had more than one child, we conducted multiple interviews with the same participant (one interview for each child). In total, we conducted 31 interviews with transgender fathers, 25 interviews with cisgender fathers who used sperm donation, and 23 interviews with cisgender fathers in natural conceptions. This represents 79 interviews.

### 2.5 Data analysis

The interviews from the Five-Minute Speech Sample (FFMS) were digitally recorded and transcribed as verbatim. A qualitative approach using thematic analysis was employed. This method was chosen to enhance the understanding of parenthood and fatherhood among transgender men in heterosexual couples with sperm donation. The qualitative method facilitated a comprehensive understanding of the phenomenon under investigation [28,29].

Initially, we adopted a general inductive approach. Each clinician red all the transcripts thoroughly, identifying the most prominent emerging themes and developing a coding framework. Then we established connections between these themes, leading to a coherent thematic organization of the interviews. This process of thematic connection involves a deeper level of analysis and interpretation to arrange the themes into meta-themes (supra-ordinate terms, according to Smith). These meta-themes describe the accounts of experiences (Smith Flowers, P., & Larkin, M., 2009). Throughout the analysis, the researcher continually cross-referenced the data and the material to ensure the coherence of the groupings. To ensure the highest possible validity of the obtained results, we analyzed the interviews twice by at least two different researchers. Each transcript was read in full by NM and LW multiple times and excerpts of text were coded in each transcript. A coding framework was developed in which coded text was examined for meaning, compared for similarities and differences, and categorized into themes and sub-themes. In addition to the qualitative analysis of the interviews, we incorporated a quantitative component to provide a clearer understanding of the prevalence of the themes discussed by participants. To achieve this, we quantified how often certain topics were mentioned by both cisgender and transgender fathers across different interviews. Tables S1, S2, and S3 (included in the supplementary materials) present the frequency and percentage of fathers and interviews that referenced specific themes and subthemes within each group (Trans-DSI, Cis-DSI, and NC groups). This quantitative breakdown allows for an understanding of the experiences shared by the participants, while maintaining the depth of the qualitative findings. All authors agreed upon final interpretations of the data. Qualitative research methods also adhere to criteria equivalent to COREQ criteria used in qualitative methods [30], which are detailed in the supplementary material. This ensures the validity and reliability of the obtained results.

## 3 Results

### 3.1 Trans-DSI group (table 1)

Fathers from the Trans-DSI group expressed mainly 3 themes related to being father, the father/child relationship and the uniqueness of the child (Table 1). ‘Being father’ is about the self-perception of fathers, and includes three subthemes related to becoming a father, to transmission, and to gender. “Father/child relationship” covers the process of establishing a bond between fathers and their children. And “Uniqueness of the child” encompasses different stages of the child's development, from the perception of their own will and desires in the present to their progressive autonomy and future projections. Table S1 (see supplementary materials) reports the occurrence (and %) of each theme and subtheme per father (N = 16) and per interview (N = 30).

**Table 1:** Meta themes and sub themes for fatherhood in Trans-DSI group (16 fathers, 31 interviews).

Being father	Father/child relationship	Uniqueness of the child
**Becoming father:**	**Sharing:**	**Today:**
Desire and father's competence	Activities	Child's will
Meeting the child	Masculine competition	At home/outside
Feeling of pride		
**Transmission:**	**Relationship with others:**	**Progress:**
Quality/imperfection	With the mother	Independence
Like me	With siblings	Projection into the future
	With the rest of the family	
**Gender:**	**Paternal love:**	
Gender preference	Need being together	
Referring to a standard	Boundary duties	

#### 3.1.1 Being father

##### 3.1.1.1 Becoming father

The first sub-theme reflects all the elements that contribute to the feeling of fatherhood. We found these elements in the narratives of 11 fathers. Three elements appear to follow a chronological logic, reflecting the father's readiness to embrace the condition of fatherhood. The first stage occurs before the child's birth, the second stage involves the almost intangible moment of the father meeting the child, and the final stage is about presenting the child to others.

*Desire and father's competence:* We observed the description of men's experiences related to their desires and doubts about their abilities with eight of the transgender men we encountered. Although our population had a higher representation of male children, we found that this question was raised to a slightly greater extent among fathers of girls compared to fathers of boys (four sons and five daughters). One father expressed that the idea of fatherhood was not something he was unable to think about: "*I never thought I would be able to have a child*”. The desire for fatherhood was actively expressed by another father, highlighting his strong inclination to have a child: "*he was more than desired*." Another father questioned his ability to support his child's educational journey, considering his own educational background and potential difficulties he might face: "*I was a little afraid, having stopped school in the third year … although self-taught, having succeeded well in my professional life, I said to myself: 'Oh, if school doesn't go well … I couldn't see myself going back to school'*.” Reflecting on his daughter, another father concluded the interview by stating: "*the … greatest gift I could have been given was to have a child*."

*Meeting the child*: The moment of meeting their child holds great significance in the fathers' narratives. It is often described as an almost fleeting moment when the father, shortly after birth, lays eyes on the child for the first time. During this brief and indescribable moment, the father's words present a timeless description (without duration, outside time). This sub them emerged in the narratives of four fathers across six interviews. One father describes this day as "*the best day, I think, of my life*." This idealized period seems to extend for this man during the first fifteen days following the child's birth. Another father, when speaking about his daughter, focuses on the precise moment of birth. He describes the birth as "*lengthy*" and recounts the meeting with the child in two stages. The first stage involves the skin-to-skin contact between the baby and the mother, observed by the father. The second stage occurs when the father observes the exercises conducted by the midwives to test the baby's reflexes. He recalls a thought that crossed his mind during this scene: "*it is when they, in quotation marks, tried to make her walk*". He reports a thought that came up to him: "*in my opinion, she is going to be fast*". Fathers in other groups seem to share this sub-theme.

*Feeling of pride*: The *feeling of pride*, though not very frequent, holds significant importance in the experience of fatherhood. We observed this sub-theme in particular with two fathers, each having only one child. One father had a little boy aged 1^1/2^, while the other had a little girl aged 4. For one of these fathers, the source of pride was his child's first words addressed to the parents. It was particularly noteworthy as the son said "*dad*" before saying "*mom*." This event was not taken lightly by the father, as he admitted: "*I was a bit very proud*". The other father expresses his pride for his only daughter in simple words: "*I am quite proud of her*". This expression of pride reflects a deep emotional connection and sense of accomplishment in his role as a father. It emphasizes the importance of the father's status to this group and the meaningful interactions he shares with his child.

##### 3.1.1.2 Transmission

The second subtheme explores the question of the ‘transmission’ of elements of the father's identity, as revealed through the descriptions of the child. It encompasses two main aspects.

*Quality/imperfection:* The father describes the child's qualities, thereby reinforcing his own identity. This implies that the father recognizes in the child certain traits that he perceives to be connected to the family unit. The tone of the fathers' descriptions of their children is in general positive, with attributes like "*affectionate*", "*kind*", "*generous*", and "*empathetic*" being commonly used. The idealization of the child is even more pronounced when the child is the only one, and the parents are divorced. One father expressed about his son: " *even his faults suit me.*" The father did not elaborate on any nuances, suggesting a strong sense of acceptance and appreciation for his child.

*Like me*: The father identifies certain characteristics in the child that he sees as reminiscent of himself, suggesting a sense of familial continuity and connection between himself – the father – and the child. One father said: "*He's not the type to express himself … to say what he feels. He tends to keep things to himself … he's a bit like me*". Another father associated the phrase "*like me*" with a significant form of concern. He described his experience with one of his children, expressing worries about the possibility of his child following a similar path as himself, a transgender journey. This father expressed concern when his son start to be more attracted to girls' toys and had more female friends than male friends. This observation led to several discussions with the child's mother. The father tried to put into perspective the experience of being transgender, acknowledging that it is not an easy journey, and he and the child's mother did not want to pass on the difficulties they had faced themselves. The father finally said: "*We don't want to transmit problems we had to bear ourselves*". These expressions suggest a strong sense of connection and recognition of shared traits, both positive and concerning.

##### 3.1.1.3 Gender

Finally, fathers from the Trans-DSI group also expressed a third subtheme related to “gender”. In French, "être du genre à" can be translates to “be of the kind” and refers to a standard. Thus this expression refers both to the question of gender, especially to the parent's preference regarding the child's gender, and a reference to the standard.

*Gender preference:*The explicit consideration of the child's gender only arises during the father's thoughts about the pregnancy and the curiosity surrounding the sex of the unborn child. This was notably observed in two fathers' narratives. One of them expressed his thoughts: "*I had cried, in fact we had both cried, and in particular I was worried, because it was going to be a boy, and so he was going to have a … a willy, whereas I didn't have one, and so I wondered how I was going to be able to manage it.*” This point appears to be unique to this particular group and was not observed in the others.

*Referring to a standard:* This was evidenced by the use of terms such as "*normal*"; "*classic*"; or "*real*" as was the case for one father. He described his child in the following manner: "*it is a true little guy, there is no problem*". Another father emphasized that his son is indeed a "*guy*" but accentuated it by adding "*true.*" This assertive statement raises questions: "*there is no problem*". This sub-theme highlights a normative dimension to fathers' representations of their children for this group.

#### 3.1.2 Father/child relationship

##### 3.1.2.1 Sharing

The first aspect involves the descriptions provided by the fathers about the activities they share together with their children. These activities play an important role in shaping the bond between the father and the child.

*Activities:* The central aspect of this sub-theme revolves around the dimension of exchanges and sharing, encompassing the activities described by the fathers. These activities can be categorized into two types: those involving care and those related to games. Example quotes: "*The bath, it's not really me, because I'm afraid … as she was small, I was afraid to break her*". This theme is also present in the Cis-DSI groups.

*Masculine competition*: ‘Sharing’ is also expressed through masculine competition. Competition did not emerge systematically during the interviews, and only two fathers explicitly addressed this issue, both in relation to their older sons: "[he] *provoke a little bit.*" This difficulty is attributed by the father to the resistance the child shows towards authority: "*it's just that sometimes … I have difficulty getting him to understand me, to accept certain things when it comes to a little authority*". The evocation of this theme never mentioned any connection with the father's gender transition path.

##### 3.1.2.2 Relationship with others

The second sub theme pertains to the communication and interaction between the father and the child, but also how this interaction requires coordination and communication with other family members, such as the mother, siblings, and extended family.

*With the mother*: Nearly half of the fathers in the cohort (seven out of sixteen) mentioned the mother in their narratives, and these mentions were present in eight interviews. The need for the father to "*find his place*" between the mother and the child was highlighted, along with the impact on the couple's life, which could be described as being "*turned upside down.*" One father described the relationship between the mother and the child as "*fusion*", emphasizing its importance. Another father also used the pronoun "*we*" to talk about the parental couple, indicating the sense of unity in their approach to parenting: "*We also have difficulty perhaps to separate from him.*"

*With siblings*: With the exception of fathers with an only child, all of the fathers in our cohort address the issue of siblings in terms of the relationship between the children and the relationship they have with each other: "*he gives his two older brothers a hard time"*; "*It's kind of folksy at home*"; "*punishments to come down like storms*". It was acknowledged that each child had different experiences in the family: "*he is the one who … who takes […] who carries […] a little bit of all the waiting"*; *"*[he] *benefits from the passage of his brother.*"

*With the rest of the family*: The child's presentation to the extended family marked the moment they assumed the role of a father for seven fathers in the study: “*Everyone wanted to come and see him … my wife's family came the next day to see him … my parents, the weekend after, because they were working … with my sister and my nephew …*”. This point underlines the importance of the recognition of the new status of being a father for the extended family in this group, more so than for the other groups.

##### 3.1.2.3 Paternal love

The third sub theme centers around the dimension of love expressed by the father towards the child. This love is intrinsically linked to the notions of fulfilling the child's needs and understanding the boundaries and separations that exist within the father/child relationship. Seven participants of this group mention their love for their child. This sub-theme is evident in the expressions of the child representing a need for the father. It encompasses the emotional bond between the father and the child, where the child becomes a source of profound love and attachment for the father and the complexities of setting boundaries.

*The need of being together* is expressed by four fathers who vividly articulate their feelings of love for their children. They describe the love they have for their child as indescribable and profound, emphasizing the strong emotional connection they feel. The father/child relationship is depicted as one of unconditional love, with the child representing a part of the father and being a significant reason for their joy and fulfillment in life. The fathers express a desire to be closely involved in their child's life, wanting to witness their growth, provide protection, and offer continuous support. Examples of expressions: "*it's … the feeling as such of love that I have for this child is quite indescribable*"; "*between us, it's a lot of father-son love … it's a lot of love … yeah, it is*"; and another father describes his relationship with the child as "*like oxygen*". In terms of frequency, this topic appears more strongly in the CIS-DSI group (see Table S1).

*Boundary duties*: This sub-theme addresses the establishment of limits within the father/child relationship and the complexities of separation. Three fathers in the study express their reflections on the difficulty of setting boundaries and separating from their children. They acknowledge that they may have been overprotective or overly nurturing, recognizing the importance of allowing their children to develop their independence and autonomy: "*It is also difficult for us to separate from him* …”; " … *it's true that we may have also nurtured him a lot* … ". This sub-theme did not occur in the other groups.

#### 3.1.3 The uniqueness of the child

##### 3.1.3.1 Today

This subtheme emphasizes the father's focus on the child's current state, their present reality, and the observations the father makes about the child's uniqueness. It involves the father's interpretation of the child's behavior and expressions of their desires and wills in the present moment.

*Child's will*: The father experiences a recognition of the child's differences and individuality, acknowledging that the child is not merely an object of parental desire but is also actively involved in constructing his/her own desires and meeting his/her needs. This recognition highlights the child's agency and autonomy, as they grow and develop their own identity and personality: "*He wants to do the right thing*"; "*they don't always have to do what is asked to them*”. A father describes his older son as having tried to "*rebel, quote-unquote, against the father's authority*". "*He knows what he wants* … *uh* … *and he knows what he doesn't want*".

*At home/Outside*: Fathers describe their children as having different aspects when *at home*, within the close family environment, and when *outside*, in different social settings. Nine fathers explicitly report differentiating between these areas for their children. E.g., “*The last one leads at home*”; “*inside the house she lets herself go a little more*”. This also underscores the father's impression that the child feels safe and secure at home, which allows him to explore the outside world.

##### 3.1.3.2 Progress

This subtheme subtly explores how fathers perceive their child's development over time. One significant aspect, observed in almost all fathers, is the child's growing autonomy. This ability for the child to express themselves and make independent choices is seen as a crucial milestone, allowing fathers to daydream about their child's future possibilities. This dynamic creates a profound and heartwarming aspect of fatherhood.

*Independence* explores the early stages of a child's independence and their differentiation from their parents. For instance, one father describes his youngest son as "*advanced*", taking "*a lot of initiative*", and displaying a strong sense of independence. This newfound independence is often associated with ideas of "*responsibility*" and "*trust*" as fathers observe their children taking on more tasks and responsibilities on their own, gaining their confidence as they explore the world around them.

*Projection into the future*: This subtheme corresponds to fathers' visions and dreams about their child's future. It involves the perspectives of seven fathers. Some express positive views, such as: "*In my opinion, later on, he will be a very happy person. He will enjoy eating, laughing, and celebrating*". "*I remember when they tried to make her walk, testing her motor skills. I thought to myself: I believe she's going to be fast. A few years later, I realized I was probably right* … " "*I might not be objective, but I think he'll grow up to be a smart little boy* … " Some fathers express more concerns: "*I hope that he will develop well into his adult life*". "*It is a child that I would like to see grow up every day, that I would like to protect, and that I would like to accompany throughout his life* … " These statements show the fathers' aspirations and anxieties about their children's future, reflecting their deep emotional investment in their roles as parents.

### 3.2 Cis-DSI group (table 2)

The cisgender DSI-group of fathers shares similar themes with the other groups such as ‘being a father’ and ‘child/father relationship’ (see Table 2). But the fathers from cisgender couples who used DSI, also evidence one unique and specific theme: ‘the gift’. This theme revolves around the concept of sperm donation and includes three sub-themes regarding ‘the donation process’, the question of ‘debt or gift’, and the ‘narrative of origins’. Table S2 (see supplementary materials) reports the occurrence (and %) of each theme and subtheme per father (N = 15) and per interview (N = 25).

**Table 2:** **Meta themes and sub-themes for fatherhood in the Cis-DSI group** (15 fathers, 25 interviews).

Parent/child relationship	Being a father	The Gift
**Sharing:**	**Becoming a father:**	**The donation process:**
Complicity	Desir	Weight of the steps
Activities	Encounter	Infertility
	Paternal love	
**The family:**	**Transmission:**	**Debt or gift:**
Roles of the father and the mother	Character and resemblance	Marital choice
Places in the family		Lack and debt
The extended family		
**Singularity of the child**	**Evolution of the relationship**	**Narrative of origins:**
Characters and interest	Rejection (fear, anticipation)	What to say?
Openness to the world	Adolescence period	When to say it?

Some cisgender fathers in this group discuss the difficulties they faced in talking about their *infertility* and the ‘donation process’. They explain the emotional *weight* and length of the procedures involved in medically assisted procreation, describing it as a long and challenging journey with uncertainties and tears. "*We waited five years* … *it was a difficult journey, there were many uncertainties, there was a lot of crying*”. "*It was a long struggle lasting several years*". They also reflect on their decisions as a couple regarding medically assisted procreation:

"*I told her yes* … *but it was extremely abstract* … *it was hard between us* … *it puts the points on the I's of what we want or not* … *I think*". Moreover, some fathers perceive their *infertility* as a lack or a weakness, feeling that they cannot physically have children like others. They express concerns about depriving their wives of a traditional pregnancy experience. "*I can't have one physically*"; "*it is an additional weakness*”; "*to have children like the others*"; "*I didn't want to deprive Lucie of being able to be a mother, to be able to have a traditional, classic pregnancy, including for me too*”. Finally, cisgender fathers focus on the *narrative of origins* thinking about how and when to talk to their children about their origins: *"We are in the middle of thinking about this* …. *Fears* … *about telling him that I am not his biological father* … *fears that come up regularly". "It's going to be just one of many topics”.* Adolescence is particularly dreaded as a moment when this topic may come up with their children: *"We start to tell him"*; *"in adolescence, I imagine it's one of the many subjects"*; *"I haven't talked about it yet, I'm waiting for the right moment when they are older”.*

### 3.3 NC group (table 3)

The cisgender group of fathers in NC are more heterogeneous and primarily focuses on two main themes: ‘child development’ and the ‘parent-child relationship’. Some fathers mention the element of chance in their pregnancy. Compared to the two previous groups (Trans and Cis-DSI), the NC group exhibits more disparity in the content of their interviews. While there are cross-cutting themes present, the topics discussed vary significantly among the participants. This group is the least homogeneous among the three, as the only common characteristic is that they are fathers who have conceived a child through natural conception. Details regarding the narrative of this group are given in Table 3. Table S3 (see supplementary materials) reports the occurrence (and %) of each theme and subtheme per father (N = 17) and per interview (N = 23).

**Table 3:** **Meta themes and sub-themes for fatherhood in the NC group** (17 fathers, 23 interviews).

Child development	Parent-child relationship
**Developmental characteristics**	**Connection between child and parents**
Descriptive aspect	Father and mother difference
	Education
**Singularity of the child**	**Relationship in the family:**
Child's behavior	Relationship in the sibling

## 4 Discussion

As previously stated, the preliminary investigation of this cohort provided us with data for the quantitative analysis of the FMSS [8]. The two-group comparisons revealed that Expressed Emotion, Criticism, and Emotional Over-Involvement were significantly higher in Trans-DSI fathers compared to both NC and Cis-DSI fathers. Notably, there was no significant difference between the Cis-DSI and NC groups concerning these emotional expressions. The aforementioned finding was discussed in the form of two hypotheses: 1) the journey of becoming a parent is a transformative experience that enriches one's identity for every individual. However, this experience differs significantly for transgender parents, leading to distinct emotional responses following their gender transition. 2) Transgender parents often navigate the expectations of a heteronormative and cisnormative society in their family-building process. They may experience anxiety in adapting to their unique circumstances, and these societal projections can influence their lived experiences and emotional expressions [8]. This new qualitative approach does not allow for an exact comparison of groups. Rather, it aims to explore the experiences of transgender fathers thanks to DSI in light of cisgender fathers. We already mentioned in the result section some similarities and differences we found. Interestingly large themes/subthemes emerged in all groups. This is the case for ‘child development’ and ‘father-child relationship’. This is reassuring as these themes are important aspects of fatherhood [16]. However, specificities also emerged for fathers who had DSI. ‘Being a father’ emerged for both cis and trans-fathers who had DSI, whereas the ‘uniqueness of the child’ emerged only for trans-DSI-fathers, and ‘the gift’ for Cis-DSI-fathers. These specificities are related to the history of fatherhood and arouse questions associated with emotions or worries. We propose to use Pessoa's concept of “*intranquility*” to refer to these emotions and worries [31]. Pessoa's original work is *Livro do desassossego*. Desassossego is a common expression in the Portuguese language. It is part of a certain habit of use and is not in itself dramatic, disturbing or even pathological. It refers to a state of inner restlessness, sometimes even intimate. Although *desassossego* has no exact translation, intranquility seems to correspond best to the idea of inwardness. This intranquility is present with two peculiarities: first, we notice that these elements come and go at several moments during the interview, as if it were something that disturbs the mind; second, when an impossible question to solve for the father occurred without any possible answer at that moment, in a way like an enigma [32]. In a phenomenological study, trans (and bisexual) parents were also found to have concerns about their children. In this respect, it appears that the best interests of the child can be a source of concern, to varying degrees, for trans parents, particularly given the challenges posed by a cisnormative environment [10], and by internalized stigma [9].

In the Trans-DSI group, fatherhood is represented as a socio-cultural aspect of masculinity. The theme of the father-child relationship highlights the father's role in helping the child discover the world and engage in physical activities. The role of the father as a separator between mother and child and the need to impose limits also create points of psychological tension for some fathers. For others, we encountered explicit reference to Oedipal issues, especially about the child who feels rivalry or competition with the same-sex parent [33]. Either transgender men inscribe their paternal identity in stereotypical references to masculinity because they would proceed from rigorous proximity to the gender role expected of fathers in Western societies [6]. Or rather, transgender men do not escape societal pressures and would consider (like other men) the necessity of meeting gender norms in order to fulfill the role of the father [34].

Fathers in the Trans-DSI group place more emphasis on the relationship between the father and the child, as well as the feeling of love that the father experiences towards the child. Physical and character resemblances are also highlighted. We note markers of ‘intranquility’ mainly observed in subthemes of gender and transmission. For example, the challenges of raising a son when the father does not have a “willy”; or worries about the child following a transgender pathway. This ‘intranquility’ is present when an impossible question to solve for the father is, in some ways, like an enigma, without any possible answer at the moment. It is something that disturbs the mind [32]. This result appears without any reference of the gamete donation in this group as the opposite for fathers from the Cis-DSI group. Moreover, this differs from results found in other qualitative studies [35]. However, in other studies, the topic of donation is brought up by the researcher as part of the interview. Here, the researchers have no influence on the topics brought up by the participants. Furthermore, one might think that our methodology prevents participants from addressing this issue, but the Cis-DSI group does.

Indeed, the Cis-DSI group is more descriptive. Fathers in this group find points of tranquility in identifying similarities between themselves and their children. However, ‘intranquility’ arises in discussions about filiation and origins, specifically related to gamete donation. Fathers express concerns about their child's reaction and how the child will understand their conception story, considering they are not the biological parent.

Regarding the NC group, the desire for a child is less insistent, and the fathers we interviewed are more diversified. While some questions about the desire for parenthood arise, it is not always the central focus for these fathers. Natural conception provides less room for questioning the desire to become parents, as the process occurs more spontaneously. The desire to be a father is met in most cases when pregnancy occurs, or because the mental load of conception (such as contraception) is held by the spouse (cisgender woman). Paterlini and coll [9]. have shown differences in the representation of the child between heterosexual couples using ART and those with natural conception. The ART group of parents have significantly more positive representations of their children than parents with natural conception. In our study, we find a positive representation of the children but less idealization than in the two other groups.

In both groups with sperm donation (Cis-DSI and Trans-DSI), fathers emphasize the existence of a strong bond and feelings of love with their children. Love is evoked and presented as of great value. Secondly, signals of ‘intranquility’ are also found in similar ways for both groups concerning the evolution of the relationship during adolescence [23]. Amongst all the interviewees, a quarter of them showed these elements. This weakening would be provoked by external factors (e.g., the child's gender, and more broadly the environment; the traditional fatherhood ideal imposed by social attitudes) that could undermine the transgender and cisgender father's gender construction hitherto established. Parents recognize that the relationship changes as the child ages, but they fear that the nature of the relationship may be challenged. It is the nature and existence of the bond, which would appear more fragile and could be threatened. In the French context, one might question the effect on a trans person of having to go through a third party to access paternity. In France, ART services, particularly gamete donation, are the exclusive domain of public assistance and the donor remains anonymous. In fact during the interviews, references to the medical service rather than the anonymous donor occurred. This involvement of a third party also involves navigating the social field. Moreover, in the French system, the financial costs are fully covered by the social security system. For transgender people, it is therefore necessary to undergo this medical examination within this public institution to request access to paternity, and thus exposing themselves to social judgment and expectations. Studies that asked trans people about their thoughts before embarking on a parenting project found that issues of expectations and the context of social normativity were important factors in their thinking [10,11].

The sperm donation pathway is more present in the discourse of cisgender fathers who have resorted to it, as well as the biological filiation. Identity linked to biology becomes a prominent concern for cisgender fathers who use DSI. In contrast, transgender individuals have already confronted this question during their transition, reflecting on the dissociation between body, experienced gender, and assigned gender at birth [27,36]. For some of them, embracing the condition of fatherhood becomes the last step of their own transition to being a man [26]. In contrast, for cisgender men who used DSI, the journey of infertility often comes as a surprise, with a brutal realization usually occurring when a desire for a child arises [37].

## 5 Limitations

The current study has several limitations. First, the care protocol at Cecos (French centers for sperm and egg donation) differed for cisgender heterosexual partners and transgender heterosexual partners. Trans heterosexual partners were required to attend an additional appointment with a psychologist, unlike heterosexual couples. Although this protocol is no longer in place today, it was for the participants in this study. Moreover, Cecos is a public institution and gamete donation is predominantly handled by public centers, with comprehensive coverage by the health system. Consequently, individuals dealing with infertility are required to undergo medical examinations within these public institutions to initiate the process of becoming parents. Requesting transgender individuals to undergo an additional psychological appointment could further undermine their confidence as parents, posing ethical concerns regarding justice [6]. Conversely, health professionals should be aware of the risks and effects of transphobia on those involved [34,35].

Second, the way we collected our Trans-DSI sample has introduced biases in the representativeness of the overall transgender population. The CECOS Cochin center receives people from all over the French metropolitan area, but it remains accessible mainly to a privileged socio-economic group of people. We only have participants in this cohort who present their gender identity in a binary way and are involved in heterosexual couples. Therefore, these results cannot be extrapolated to other situations of transgender affirmation or non-binary gender identities.

Third, we cannot exclude motivational and social desirability biases. Transgender men who participated in our research might have adjusted their responses to align with what they perceived as meeting our desired outcome [20]. In addition, participants may have adjusted their responses to align with perceived societal norms, especially in a sensitive topic like this. As a result, transgender fathers may have been inclined to present their families as well-adjusted, showing that their children are developing positively and that their families are not significantly different from others. This consideration could have influenced the way they expressed themselves during our interviews.

Fourth, it is important to acknowledge methodological limitations: qualitative studies do not allow us to draw definitive conclusions about similarities or differences between the samples as they include a number of participants rather limited. Our primary goal was to gain a representation of fatherhood experiences in different modes of birth conception. From this perspective, one could argue that another group could have been included to increase meaningfulness and diversity of experiences: cisgender fathers with IVF but without DSI. Not having this comparison group is a limitation, as it could have provided valuable insights into the specific impact of ART on fatherhood experiences. Also, FMSS is a standardized interview with good validation. However, the 5 min duration and the specific instructions may not capture the full spectrum of family dynamics, as it primarily focuses on expressed emotions towards the child and may not address broader family interactions or conflicts.

Finally, regarding our analyses, we could have involved transpersons in the research group [29]. As all members of our research team are cisgender individuals, not having individuals who have experienced the phenomenon of transition could be considered an important limitation. However, we had the opportunity to present our results during conferences, where some participants of this study were present, and received feedback from them. It appears that the concept of ‘intranquility’ resonated with them and was considered relevant [38].

## 6 Conclusion

There are various ways of becoming a father, each with shared emotions and concerns common to all fathers, as well as other more specific ones depending on the conception history and parenthood journey of each parent. This study is among the few that specifically explore the post-ART experiences of transgender parents, with a particular focus on their lived parental experiences. Addressing a significant gap in the current literature, which predominantly centers on the pre-parenting phase, our work offers a important contribution to advancing the understanding of transgender parenthood after ART. For transmen who access parenthood through DSI, fatherhood is often defined within the social role of masculinity, emphasizing involvement in physical activities and helping the child explore the world and his future. Some fathers express intimate *intranquility* in their role and the future of their children. We believe it is important to adapt the support provided to fathers based on the specificities related to their pathways to parenthood, especially if they have experienced infertility. In such cases, it becomes essential to consider the underlying cause of infertility and the journey they had to go through. This reconfiguration period can challenge a father's self-confidence, particularly when he is a transgender father and/or does not have a genetic link with his child. In other situations, some trans fathers experiencing pregnancy tend to avoid hospitals to reduce the risk of transphobia [39]. When external factors collide with intimate elements, it can lead to psychological tension for the father.

Finally, we believe that this research as two main implications regarding support and research. First, healthcare professionals should receive appropriate training in providing support [4,40]. Additionally, peer support could enable individuals to draw on community knowledge, which is effective in overcoming barriers to sexual health prevention [41] and serving as a protective factor against suicide risk [42]. Providing support to parents from conception to the pivotal period of adolescence, when questions about relationships may resurface, can be done through father support groups or by developing educational resources that are rarely available to non-cisgender parents-to-be. Second, future research should explore to what extent the intranquility might be related to experiences of minority stress, which is this chronic stress experienced by individuals belonging to stigmatized minority groups, due to social prejudice, discrimination, and marginalization [43]. Also, in the recent years, new ways of accessing fatherhood for trans individuals has occurred (e.g., pregnancy in transgender men who kept their uterus by stooping masculinizing hormones). How this journey affects fatherhood should also be investigated.

## CRediT authorship contribution statement

**N. Mendes:** Writing – original draft, Validation, Project administration, Methodology, Investigation, Funding acquisition, Formal analysis, Data curation, Conceptualization. **L. Woestland:** Writing – original draft, Validation, Methodology, Formal analysis. **V. Drouineaud:** Validation, Methodology, Investigation, Conceptualization. **F. Poirier:** Validation, Methodology, Investigation. **C. Lagrange:** Validation, Investigation. **O. Rosenblum:** Validation, Investigation. **J.-P. Wolf:** Validation. **C. Patrat:** Validation, Supervision. **J. Brunelle:** Validation. **F. Pommier:** Validation, Supervision. **D. Cohen:** Validation, Supervision, Conceptualization. **A. Condat:** Validation, Supervision, Investigation, Conceptualization.

## Data availability statement

The detailed data that has been used is confidential and cannot be shared due to ethical requests.

## Declaration of competing interest

The authors declare the following financial interests/personal relationships which may be considered as potential competing interests: Nicolas Mendes reports financial support was provided by Pfizer Foundation. Other authors declare that they have no known competing financial interests or personal relationships that could have appeared to influence the work reported in this paper.

## Footnotes

1 *Centre d’Etudes et de Conservation des Œufs et du Sperm* (CECOS): Center for Egg and Sperm Studies and Conservation.
