# Supplementary material for: Fatherhood experiences: A qualitative approach of cisgender and transgender fathers in assisted reproductive technologies (ART) situation with sperm donation
Source: Heliyon. 2024 Nov 16;10(22):e40501. doi: 10.1016/j.heliyon.2024.e40501 (PMC11625155; doi:10.1016/j.heliyon.2024.e40501)
Supplement: Multimedia component 1 [file mmc1.docx]

**Research team and reflexivity (COREQ):**

**32-item checklist**

Personal Characteristics

Interviewer/facilitator: Nicolas Mendes

Credentials: PhD student

Occupation: research psychologist

Gender: male

Experience and training: was trained in qualitative analysis by a team of qualitative researchers (Sibeony, et al.)

Relationship with participants

Relationship established: no

Participant knowledge of the interviewer: The participants knew that the aim of the study was to explore the reality of trans parenthood.

Interviewer characteristics: the researcher was a white cisgender gay man who approach this topic with respect and sensitivity towards transgender individuals

**Theory**
Participant selection

9. Methodological orientation and Theory: general inductive approach, phenomenology

10. Sampling: from Cochin CECOS CENTER in Paris, France
11. Method of approach: email and telephone
12. Sample size: 48 participants
13. Non-participation: 10 participants

Setting

14. Setting of data collection: Data collection took place in a clinical setting
15. Presence of non-participants: No non-participants were present during data collection

16. Description of sample: The sample consisted of individuals from socio-economically privileged backgrounds.

Data collection

17. Interview guide: the FMSS instruction
18. Repeat interviews: No repeat interviews were conducted.
19. Audio/visual recording: Data collection involved only audio recordings, with no visual recording.
20. Field notes: yes
21. Duration: Each session lasted approximately 20 minutes, including 5 minutes for the interview recording and additional time for preparation and debriefing.
22. Data saturation: yes
23. Transcripts returned: no

**Analysis and finding**

Data analysis

24. Number of data coders: 2
25. Description of the coding tree: A coding tree was developed to organize the data.

26. Derivation of themes: derived from the data
27. Software: no software used
28. Participant checking: yes

Reporting

29. Quotations presented: Quotations from participants were presented in the findings.

30. Data and findings consistent: yes

31. Clarity of major themes: yes
32. Clarity of minor themes: yes
