# Supplementary material for: Fatherhood experiences: A qualitative approach of cisgender and transgender fathers in assisted reproductive technologies (ART) situation with sperm donation
Source: Heliyon. 2024 Nov 16;10(22):e40501. doi: 10.1016/j.heliyon.2024.e40501 (PMC11625155; doi:10.1016/j.heliyon.2024.e40501)
Supplement: Multimedia component 2 [file mmc2.docx]

**Supplementary Materials**

**Table S1. Trans-DSI Group : number (%) of fathers and number (%) of interviews that reported a given theme and subtheme during FMSS interviews**

| Theme | Number of fathers | %  fathers | Number of interviews | %  children |
| --- | --- | --- | --- | --- |
| **Being father** | **16** | **100** | **30** | **96,8** |
| *Becoming father* | *11* | **68,8** | *13* | **41,9** |
| Desire and father's competence | 8 | **50** | 10 | **32,3** |
| Meeting the child | 4 | **25** | 6 | **19,4** |
| Feeling of pride | 2 | **12,5** | 2 | **6,5** |
| *Transmission* | *11* | **68,8** | *24* | **77,4** |
| Quality/imperfection | 11 | **68,8** | 19 | **61,3** |
| Like me | 8 | **50** | 12 | **38,7** |
| *Gender* | *5* | **31,3** | *8* | **25,8** |
| Gender preference | 2 | **12,5** | 3 | **9,7** |
| Referring to a standard | 4 | **25** | 5 | **16,1** |
| **Father/child relationship** | **16** | **100** | **31** | **100** |
| *Sharing* | *11* | **68,8** | *18* | **58,1** |
| Activities | 10 | **62,5** | 17 | **54,8** |
| Masculine competition | 2 | **12,5** | 2 | **6,5** |
| *Relationship with others* | *11* | **68,8** | *21* | **67,7** |
| With the mother | 7 | **43,8** | 8 | **25,8** |
| With siblings | 2 | **12,5** | 2 | **6,5** |
| With the rest of the family | 9 | **56,3** | 17 | **54,8** |
| *Paternal love* | *7* | **43,8** | *9* | **29** |
| Need being together | 4 | **25** | 5 | **16,1** |
| Boundary duties | 3 | **18,8** | 4 | **12,9** |
| **Uniqueness of the child** | **16** | **100** | **23** | **74,2** |
| *Today* | *12* | **75** | *16* | **51,6** |
| Child's will | 8 | **50** | 8 | **25,8** |
| At home/outside | 9 | **56,3** | 11 | **35,5** |
| *Progress* | *11* | **68,8** | *14* | **45,2** |
| Independence | 6 | **37,5** | 8 | **25,8** |
| Projection into the future | 7 | **43,8** | 7 | **22,6** |
| **Total cohort** | 16 | **100** | 31 | **100** |

**Table S2. Cis-DSI Group: number (%) of fathers and number (%) of interviews that reported a given theme and subtheme during FMSS interviews**

| Theme | Number of fathers | %  fathers | Number of interviews | %  children |
| --- | --- | --- | --- | --- |
| **Being father** | **15** | **100** | **24** | **96** |
| *Becoming father* | *14* | **93,3** | *23* | **92** |
| Desir | 8 | **53,3** | 15 | **60** |
| Encounter | 5 | **33,3** | 10 | **40** |
| Paternal love | 13 | **86,7** | 21 | **84** |
| *Transmission* | *13* | **86,7** | *18* | **72** |
| Character and resemblance | 13 | **86,7** | 18 | **72** |
| *Evolution of the relationship* | *13* | **86,7** | *15* | **60** |
| Rejection (fear, anticipation) | 11 | **73,3** | 13 | **52** |
| Adolescence period | 4 | **26,7** | 4 | **16** |
| **Parent/child relationship** | **15** | **100** | **25** | **100** |
| *Sharing* | *13* | **86,7** | *23* | **92** |
| Complicity | 11 | **73,3** | 17 | **68** |
| Activities | 10 | **66,7** | 20 | **80** |
| *The family* | *14* | **93,3** | *23* | **92** |
| Roles of the father and the mother | 10 | **66,7** | 17 | **68** |
| Places in the family | 9 | **60** | 16 | **64** |
| The extended family | 5 | **33,3** | 5 | **20** |
| *Singularity of the child* | *14* | **93,3** | *23* | **92** |
| Characters and interest | 13 | **86,7** | 21 | **84** |
| Openness to the world | 7 | **46,7** | 11 | **44** |
| **The Gift** | **13** | **86,7** | **15** | **60** |
| *The donation process* | *8* | **53,3** | *11* | **44** |
| Weight of the steps | 8 | **53,3** | 9 | **36** |
| Infertility | 7 | **46,7** | 10 | **40** |
| *Debt or gift* | *7* | **46,7** | *9* | **36** |
| Marital choice | 6 | **40** | 7 | **28** |
| Lack and debt | 5 | **33,3** | 6 | **24** |
| *Narrative of origins* | 7 | **46,7** | 8 | **32** |
| What to say? | 7 | **46,7** | 8 | **32** |
| When to say it? | 5 | **33,3** | 5 | **20** |
| **Total cohort** | 15 | **100** | 25 | **100** |

**Table S3. NC group: number (%) of fathers and number (%) of interviews that reported a given theme and subtheme during FMSS interviews**

| Theme | Number of fathers | %  fathers | Number of interviews | %  children |
| --- | --- | --- | --- | --- |
| **Child development** | **17** | **100** | **23** | **100** |
| *Developmental characteristics* | *15* | **88,2** | *19* | **82,6** |
| Descriptive aspect | 13 | **76,5** | 16 | **69,6** |
| *Singularity of the child* | *10* | **58,8** | *17* | **73,9** |
| Child's behavior | 14 | **82,3** | 18 | **78,3** |
| **Parent/child relationship** | **17** | **100** | **23** | **100** |
| *Connection between child and parents* | *12* | **70,6** | *21* | **91,3** |
| Father and mother difference | 11 | **70,6** | 20 | **86,9** |
| Education | 9 | **52,9** | 19 | **82,6** |
| *Relationship in the family* | *14* | **100** | *20* | **86,9** |
| Relationship in the sibling | 9 | **82,3** | 15 | **65,2** |
| **Total cohort** | 17 | **100** | 23 | **100** |
